# Comparative analysis of Feedlot and Free-range cattle management on Botswana beef quality: a study of sustainability, post-mortem examination, and SWOT analysis

**DOI:** 10.1007/s11250-025-04355-1

**Published:** 2025-03-10

**Authors:** Brianna E. Parsons, Joshuah Makore, Boisy Motube, Gregory Thato Rakobe, Solomon Stephen Ramabu

**Affiliations:** 1 School of Veterinary Medicine, University of Pennsylvania, Center for Stewardship Agriculture and Food Security, New Bolton Center, Kennett Square, PA USA; 2 FAIR Farms Gambia, https://www.fairfarmsgambia.com; 3 Department of Veterinary Sciences, Content Farm, Botswana University of Agriculture and Natural Resources, Gaborone, Botswana; 4 Multispecies Abattoir Botswana, Gaborone, Botswana

**Keywords:** One Health, Climate change, African animal agriculture, Sustainable development goals, Mixed-method approach

## Abstract

African agriculture faces triple threats of climate change, population growth and inequality, necessitating sustainability research in agriculture that comprehensively examines social, economic, environmental, and socio-political trade-offs. To examine sustainable beef production in Botswana, this study used a mixed-method approach to explore Feedlot and Free-range cattle management systems. First, competing but overlapping factors of environmental, economic, and social sustainability in beef production were identified from literature review, and a conceptual framework for sustainability was used to assess the trade-offs inherent in both management systems. Second, abattoir post-mortem examinations were used to compare 25 feedlot cattle and 22 free-range cattle across standard factors including: carcass dress mass (CDM), carcass grade, fat colour, conformation, dentition, carcass and offal postmortem decisions, and reasons for the decisions. Lastly, sustainability trade-offs for feedlot and free-range cattle management were compared through a Strengths, Weaknesses, Opportunity, and Threats (SWOT) analysis. Sustainability assessment showed that feedlots rely on imported grains like corn and soybean, increasing producers’ uncertainty as an increasingly turbulent world affects grains availability and pricing. Free-range pasture systems, on the other hand, rely on rainfed agriculture (and often communal lands), which are increasingly challenged by the intersections of climate change, overgrazing and issues of land access. Feedlot animals had a significantly higher CDM (*p = *0.0001) at 237.56 kg ± 6.98 (Mean ± SEM) compared to 184.50 kg ± 7.44 for Free-range cattle and higher incidences of red offal condemnation rates (lung, liver, and spleen) (*p* < 0.05) compared to free-range cattle. Free-range cattle had higher green offal condemnation rates specifically large tripe (*p* = 0.026) compared to feedlot cattle. SWOT analysis confirmed the trade-offs in several factors including carcass weight, feed costs, and climate change impacts. The conceptual framework for sustainability, postmortem findings and SWOT analysis reveal cattle farming challenges and trade-offs that must be made to address disease burdens, animal nutrition, and productivity in Botswana.

**Supplementary Information:**

The online version contains supplementary material available at 10.1007/s11250-025-04355-1.

## Introduction

African agriculture and food systems must produce more food for a growing population (Ritchie et al. 2023; United Nations 2023) with environments under threats of climate change. Botswana, like much of Southern Africa, is expected to experience the highest increases in temperature, along with projections of 10% decline in rainfall by 2025 due to climate change (Akoon et al. 2011; IPCC 2014). The globally renowned Botswana beef sector encompasses over 1.7 million head of cattle (International Trade Administration 2024) and holds the ninth largest market share of countries exporting beef to the European Union (Chatibura 2023). Cattle rearing was the backbone of Botswana’s economy in the pre-colonial period and remains a critical part of the rural economy, subject to government investment over the last decades to develop infrastructure, marketing, and veterinary services in support of the sector (Darkoh and Mbaiwa 2002). Livestock production, and especially cattle rearing, contributes an estimated 80% of agricultural GDP (International Trade Administration 2024). *Seswaa* (pounded beef), the national dish of Botswana, pays tribute to the unique taste of Botswana beef as well as the Setswana culture of cattle raising across the savanna, bush, and semi-arid landscape of the country. How the cultural, environmental, and economic systems of Botswana’s beef production sustainably respond to a changing climate is the subject of this study.

Beef production in Botswana occurs in two distinct management systems – Feedlot and Free-range. These systems have significant differences in: animal feeding, land use, animal health, ecosystem impacts, and productivity. Feedlot management intensively manages animals, bringing concentrate grain feeds to the animals held in smaller, typically enclosed paddocks. Concentrate grains grown domestically (sorghum, maize, millet, Tswana cowpeas) often do not meet domestic demand, especially in years of drought (International Trade Administration 2024), causing cereal grains to be Botswana’s largest food import, totalling upwards of 23% of all total food importation (~ $14.5 million USD) through sourcing regionally (Zimbabwe, South Africa) and internationally (Brazil, Australia) (Coleman 2024; Statistics Botswana 2022). In traditional Free-range systems, or more extensive management, pasture grazing predominates, typically on communally held non-fenced land. Free-range management accounts for over 80% of the total cattle population (Darkoh and Mbaiwa 2002), while commercial production represents 1% of all farms, holding 8% of total farmland area, and producing 20% of the country’s cattle (FAO 2005; International Trade Administration 2024; USAID LandLinks 2024). Some attribute the unique taste of Botswana beef to the nutrition of free-range pastured cattle consuming the native and indigenous plants of the nation (Sunday Standard Reporter 2023), highlighting the One Health linkages between culture, environments, and human nutrition within agriculture. Beef export sales to the E.U. are facilitated by the Botswana Meat Commission (BMC), a parastatal-run abattoir and marketing body (Darkoh & Mbaiwa 2002). Both Feedlot and Free-range systems experience impacts of climate change, namely droughts and increased temperatures, but the landless Free-range farmers relying on communal lands, often lacking pasture management strategies, suffer the most intense consequences of both a changing climate and the ‘tragedy of the commons’ through overgrazing of cattle.

This study sought to understand sustainability of Feedlot and Free-range cattle management systems in Botswana by deploying a mixed-method approach. As a systems concept, sustainability research benefits from mixed-methods research which, by design, integrates evidence across interconnected disciplines. Sustainable agriculture is influenced by various disciplines including: animal nutrition and productivity, ecosystem health, public health and nutrition, and global food and feed trade. Traditional African animal agricultural research typically focus on one discipline. Despite the inherent interconnectivity, there is a paucity of conceptual frameworks that holistically assess sustainable cattle management systems in African countries, and specifically Botswana. Similarly, while data availability, collection and costs can limit agricultural research across African countries, the use of existing, routinely collected data to conduct country-specific research is limited. In this case, the research team evaluated quantitative, routine abattoir post-mortem examination information. The abattoir serves the livestock sector through disease surveillance and public health through removal of meat that is not fit for human consumption from the food chain. Abattoir level data was used to understand real sustainability outcomes of Botswana beef production, examining routinely collected information including weight, age, fat colour, meat grade, and carcass and organ condemnation rates for 47 cattle carcasses. Using mixed-methods and pairing country-level data with qualitative information and lived experiences through a SWOT analysis (strengths, weaknesses, opportunities, threats) allows greater complexity and specificity in identifying sustainability variables, challenges, trade-offs and implications for Botswana’s agricultural sector at large. The outcome of the mixed-method approach employed in the study is discussed highlighting the differences and similarities between the two cattle management systems.

## Materials and methods

### Botswana beef sector conceptual framework on sustainability

Sustainability, as a systems concept of complexly interrelated parts (i.e. social, cultural economic, environmental, geopolitical factors) across time scales (i.e. present and future), benefits from frameworks to describe issues with clarity and objectivity. This is particularly important when comparing agricultural production and management systems. Adapting concepts, principles and definitions as reviewed by Ruggerio (2021), a conceptual framework for sustainability was created to facilitate sustainability comparisons between feedlot and free-range cattle management systems. Qualitative information from literature review and lived experiences are used to describe sustainability considerations across the two management systems, considering the divergent themes of both food security and food sovereignty. Food security, defined at the 1996 World Food Summit, is when all people at all times have physical and economic access to sufficient safe and nutritious food that meets their dietary needs and food preferences for an active and healthy life, concerned with factors of food availability, accessibility, utilization and stability (World Bank 2024). Food sovereignty, first defined by La Via Campesina in 1996 and codified in the Declaration of Nyéléni in 2007, defines the right of all peoples to culturally appropriate, healthy, and nutritious foods, as well as the right of ownership of agricultural production systems (La Via Campesina 1996, 2008; Nyeleni Village 2007). Both approaches are considered in sustainability assessments.

### Comparison of Feedlot with Free-range cattle at postmortem

Routine abattoir post-mortem records were collected from the Botswana Multispecies Abattoir over May 2023 to facilitate comparison between groups of cattle raised in Feedlot and Free-range management. An observational study was performed in which two batches of slaughter animals consisting of 25 feedlot cattle from the same farm and 22 free-range cattle from the same communal holding were randomly selected. Data from postmortem reports of the two batches was entered into a Microsoft Excel spreadsheet. The data included: management type, carcass dress mass, sex, fat colour, conformation, dentition, carcass and offal detention, and reasons for detention. The un-paired t-test which is appropriate for comparing two group means of quantitative variables was applied in the separation of the means. The animals were not grouped or paired hence the unpaired t-test. The chi-square test, relevant in testing categorical data, was used to test the following qualitative variables: grade, fat colour, dentition, and reasons for post-mortem decisions.

### Strengths, Weaknesses, Opportunity, and Threats (SWOT) analysis of Botswana beef sector

A Strengths, Weaknesses, Opportunity, and Threats (SWOT) analysis of Botswana beef sector was carried out comparing qualitative sustainability factors from literature and quantitative abattoir meat quality findings on feedlot and free-range cattle management. Cattle management system considerations fell across social, economic, environmental, and geopolitical factors, including factors such as: cattle productivity, cattle nutrition, system profitability, feed costs and global trade, reliability/volatility of feed source, resilience during global turbulence, animal health and disease conditions, and present and future impacts of climate change. 

## Results

### Botswana beef sector conceptual framework on sustainability

**Fig. 1:**
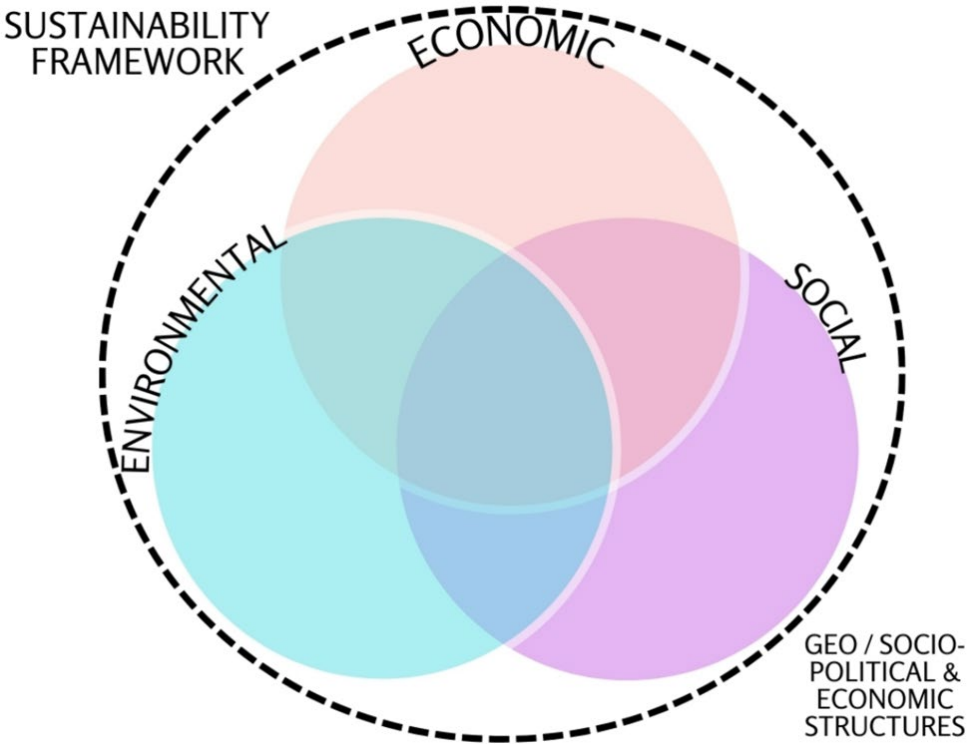
Sustainability diagram showing competing but overlapping factors of environmental, economic, and social sustainability all influenced by geo- and socio-political and economic structures. Adapted from (Ruggerio 2021)

By examining literature on sustainability, and in accordance with Ruggerio 2021, a conceptual framework was developed for sophisticated comparison of sustainability trade-offs (Fig. 1). Applying this framework to Botswana’s beef sector allowed examination of social, economic, and environmental decisions and trade-offs, all constrained by geo- and socio-political and economic structures. Farm management decisions necessitate sustainability trade-offs, which are determined both by values, norms, economics, and power structures. Cattle feeding, for example (i.e. feedlot or free-range), is an important sustainability decision – influenced by economic and environmental factors, and resulting in threats, challenges, and opportunities for each system. Feedlots rely on imported grains like corn and soybean, increasing producers’ uncertainty as an increasingly turbulent world affects grains availability and pricing. Free-range pasture systems, on the other hand, rely on rainfed agriculture (and often communal lands), which are increasingly challenged by the intersections of climate change, overgrazing and issues of land access. The sustainability framework in Fig. 1 facilitates detailed understandings of sustainability differences across Botswana’s beef management systems.

### Comparison of Feedlot with Free-range cattle at postmortem

The effect of finishing beef cattle in Feedlot as compared to Free-range was determined by comparing abattoir postmortem findings of the two groups of animals. There was a significant difference (*p* = 0.0001) between the carcass dressed mass (CDM) of cattle from the Feedlot compared to cattle from Free-range with Feedlot cattle weighing on average 53 kg more (Table 1). Feedlot cattle attained a higher grade of prime significantly (*p* = 0.0001) more than Free-range cattle that were graded grade 1 or grade 2 but not prime. Factors that affect grade are age which is determined by dentition, conformation, and fat colour. There was no significant difference (*p* = 0.1634) in dentition between the two groups of cattle. For Free-range cattle 64% had 0 teeth, 27% 1–6 teeth, and 9% 8 teeth, whereas for Feedlot cattle 84% had 0 teeth, 16% 1–6 teeth, and 0% 8 teeth. Feedlot cattle were significantly (*p* = 0.0001) different from Free-range cattle for fat colour and carcass conformation (Table 2). The former had 100% white fat whilst the latter had 59% no fat, 27% coloured fat, and 14% white fat. Feedlot cattle had 100% good conformation and free-range cattle had 23% poor, 50% fair and 27% good conformation. None of the carcasses in the current study were condemned. The heart of one Feedlot animal was condemned for infestation with *Cysticercus bovis* (Table 3). There were no significant differences (*p* = 0.2375) in the number of carcasses, heads, hearts, and tongues that were detained between the two groups of cattle. The sole reason for detention across management types was infestation with beef measles (*Cysticercosis bovis*).

**Table 1:** Carcass dressed mass (Mean ± standard error of the mean) and frequencies of grade and dentition for Feedlot and Free-range cattle

Parameter	Management type		
	Feedlot	Free-range	*P*-value
Carcass Dressed Mass (CDM) (kg ± s.e.m*)	237.56 ± 6.98	184.50 ± 7.44	< 0.0001
	No. of Cattle (Percentage)		
Grade			
1	0 (0)	17 (77)	< 0.0001
2	0 (0)	5 (23)	
Prime	25 (100)	0(0)	
Dentition			
0 tooth	21 (84)	14 (64)	0.1634
1–6 teeth	4 (16)	6 (27)	
8 teeth	0 (0)	2 (9)	

*s.e.m = standard error of the mean

**Table 2:** Frequencies of fat colour and carcass conformation for Feedlot and Free-range cattle

Parameter	Management type		
	Feedlot	Free-range	*P*-value
Fat colour	No. of Cattle (Percentage)		
None*	0 (0)	13 (59)	< 0.0001
Cream or yellow	0 (0)	6 (27)	
White	25 (100)	3 (14)	
Carcass Conformation			
Poor	0 (0)	5 (23)	< 0.0001
Fair	0 (0)	11(50)	
Good	25 (100)	6 (27)	

* none = carcasses that were emaciated and had no fat and hence the colour cannot be described

**Table 3:** Frequencies of postmortem decision of carcass, head, tongue, and heart for Feedlot and Free-range cattle

Parameter	Management type		
	Feedlot	Free-range	*P*-value
Carcass post-mortem	No. of Cattle (Percentage)		
Detained	1 (4)	3 (14)	0.2375
Passed	24 (96)	19 (86)	
Reason for detention of Carcass			
* C. bovis*	1 (4)	3 (14)	0.2375
None	24 (96)	19 (86)	
Head post-mortem			
Detained	1 (4)	3 (14)	0.2375
Passed	24 (96)	19 (86)	
Reason for detention of Head			
* C. bovis*	1 (4)	3 (14)	0.2375
None	24 (96)	19 (86)	
Tongue post-mortem			
Detained	1 (4)	3 (14)	0.2375
Passed	24 (96)	19 (86)	
Reason for detention of Tongue			
C. bovis	1 (4)	0 (0)	0.3430
None	24 (96)	22 (100)	
Heart post-mortem			
Condemned	1 (4)	0 (0)	0.3331
Detained	1(4)	3 (14)	
Passed	23 (92)	19 (86)	
Reasons for post-mortem decision of Heart			
Contamination	1 (4)	0 (0)	0.3331
C. bovis	1 (4)	3 (14)	
Passed	23 (92)	19 (86)	

* C. bovis = *Cysticercosis bovis*, or beef measles

**Fig. 2:**
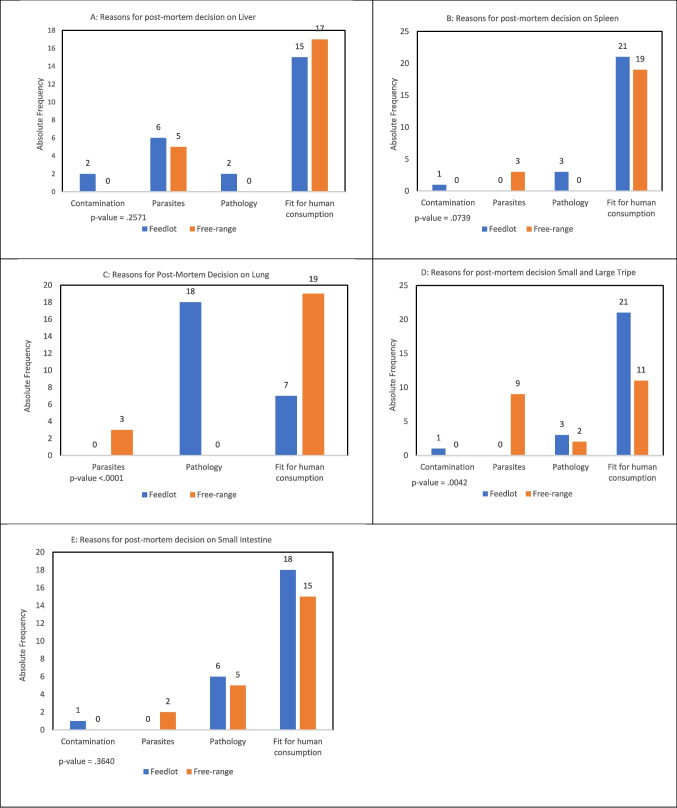
Reasons for organ post-mortem decisions across Feedlot and Free-range management systems. Abattoirs routinely inspect all organs and render a decision whether they are fit for human consumption (passed), or unfit for human consumption (condemned), or should be detained for further investigation. **A**: Liver, **B**: Spleen,** C**: Lung, **D**: Small and Large Tripe,** E**: Small Intestine

The outcome of offal inspection is condemnation, detention, or being passed as fit for human consumption (Table 4). The decision to condemn organs differed (p < 0.05) between the two management groups for all organs except small intestine. Notably, Feedlot cattle had higher incidences of red offal condemnation rates (lung, liver, and spleen) (p < 0.05) compared to free-range cattle whereas Free-range cattle had higher green offal condemnation rates specifically small and large tripe (*p = *0.026) compared to feedlot cattle. Significantly more livers of Feedlot cattle were condemned (*p = *0.0158) at 40% than those of free-range cattle at 9% (Table 4, Fig. 2A). Contamination and pathology were reasons for either condemnation or detention of livers in feedlot and not in free-range cattle. Spleens of Feedlot cattle were condemned significantly more at 16% (*p = *0.0314) than those of free-range cattle at 0% (Table 4, Fig. 2B). Condemnation of Feedlot cattle spleens was attributable to contamination and pathology whereas in Free-range cattle the reason was parasites. Lungs of Feedlot cattle were condemned significantly more at 72% (*p = *0.0001) than those of Free-range cattle at 0% (Table 4, Fig. 2C). Condemnation of lungs was attributable to pathology for Feedlot whereas detention of Free-range cattle lungs was attributable to parasites.

**Table 4:** Frequencies of postmortem decision for liver, spleen, lung, small and large tripe, and small intestine for Feedlot and Free-range cattle

Parameter	Management type		
	Feedlot	Free-range	*P*-value
Liver post-mortem	No. of Cattle (Percentage)		
Condemned	10 (40)	2 (9)	0.0158
Detained	0 (0)	3 (14)	
Passed	15 (60)	17 (77)	
Spleen post-mortem			
Condemned	4 (16)	0 (0)	0.0312
Detained	0 (0)	3 (14)	
Passed	21 (84)	19 (86)	
Lung post-mortem			
Condemned	18 (72)	0 (0)	< 0.0001
Detained	0 (0)	3 (14)	
Passed	7 (28)	19 (86)	
Small & Large Tripe post-mortem			
Condemned	4 (16)	8 (36)	0.0260
Detained	0 (0)	3 (14)	
Passed	21 (84)	11 (50)	
Small intestine post-mortem			
Condemned	7 (28)	5 (23)	0.2975
Detained	0 (0)	2 (9)	
Passed	18 (72)	15 (68)

Small and large tripe of Free-range cattle were condemned significantly more at 36% (*p = *0.0260) than tripe of Feedlot cattle at 16% (Table 4, Fig. 2D). The reasons for condemnation varied with tripe from free-range cattle being condemned mostly for parasites and less so for pathology whereas feedlot cattle tripe was condemned mostly for pathology and less so for contamination and none for parasites. Thus, parasites were a much more important factor in Free-range cattle compared to Feedlot cattle in determining condemnation of tripe. Condemnation of small intestines did not differ (*p = *0. 2975) between the cattle groups (Table 4, Fig. 2E). Condemnation was attributable to contamination and pathology in feedlot cattle whereas parasites and pathology were responsible for condemnation in Free-range cattle.

### Strengths, Weaknesses, Opportunity, and Threats (SWOT) analysis of Botswana beef sector

Sustainability considerations for the Botswana beef sector were classified according to a SWOT analysis, presented in Table 5. The SWOT analysis compliments the sustainability framework in Fig. 1, by highlighting animal management and sustainability trade-offs between Feedlot and Free-range management. SWOT analysis identified higher carcass weights, production efficiency, and stable nutrition as strengths for the Feedlot system and low feed costs and reliance on native feeds as strengths for the Free-range system. Weaknesses of Feedlot included reliance on imported feeds and high rates of organ condemnation, compared to Free-range weaknesses of lower carcass weights impairing profitability and productivity of the management system. Opportunities diverged across the two systems, where Feedlot systems can benefit from reducing disease conditions and developing resilient feed sourcing methods. Free-range systems have opportunities in developing sustainable pasture management strategies to improve nutrition and production of Free-range cattle, as well as developing marketing strategies for grass-fed products. Both systems are threatened by climate change exacerbating animal disease, heat stress, and declining animal welfare.

**Table 5:** Strength, Weakness, Opportunity, and Threat (SWOT) analysis for Feedlot and Free-range cattle management in Botswana

	Feedlot	Free-range
Strengths	- Large carcass weight = higher revenues, more meat produced - Production efficiency economic model effective under current economic systems - Stable cattle nutrition compared to pasture systems	- Low feed costs - Relies on native feeds ➜ food sovereignty aligned resiliency through self-sufficiency in feed production
	- Botswana Government offers various supports for farmers across cattle production systems, such as subsidies, grants, and veterinary services	
Weakness	- Reliance on imported feed: ○ Potential volatility in availability and pricing affecting feed costs - High rates of organ condemnation	- Lower carcass weight, due to: ○ Declining pasture qualities (overgrazing and/or climate change) ○ Evidence of parasitisation at abattoir (result of declining pasture qualities and/or overstocking/overgrazing of communal pastures) - Smaller carcass weights = lower revenues, less meat produced
Opportunities	- Reduce disease conditions in feedlot ○ Trade-off consequences with productivity, as methods to reduce disease conditions often decrease productivity (ex. reduce crowding or overstocking to reduce infectious disease pressures like respiratory disease) - Improve resiliency of feed sourcing	- Pasture management strategies ○ Regenerative / strategic grazing practices to restore soil health and pasture quality ○ Managing drought tolerant species for climate change resilience ○ Research for improving forage quality ○ Communal land access and management policies - Market segments demanding organic and grass-fed beef
Threats	- Turbulent global markets impacting grain importation, access and/or pricing - Climate change impacting health of intensively managed animals at higher risk of disease spread (ex. infectious and zoonotic diseases, heat stress at higher intensities in intensified management conditions)	- Climate change causing continued pasture degradation - Climate change impacting health of animals (heat stress, infectious/ zoonotic disease) - Vulnerability to predators and theft

## Discussion

This study examined meat quality from Botswana’s beef sector, an intangible cultural heritage of the nation, by comparing management practices of Feedlot and Free-range beef production and examining sustainability trade-offs. Animal productivity, animal disease burden, and beef sector profitability all exist on an interactive continuum, creating trade-offs dependent on the management system (Breure et al. 2024; Kanter et al. 2018; Thomson et al. 2019). African Agriculture, including the beef sector, needs to be sustainable. Our assessment concurs with Ruggerio 2021, recognizing sustainability as the outcome of competing but overlapping factors of environmental, economic, and social nature all influenced by geo- and socio-political and economic structures. This conceptual framework was validated by quantitative postmortem findings and qualitative findings from SWOT analysis. Together, this mixed-method approach highlighted significant sustainability differences between Feedlot and Free-range beef management practices across economic (i.e. Feedlot systems experiencing higher returns from higher carcass weights), environmental (i.e. both feeding strategies threatened by climate change in different ways), social (i.e. Free-range systems a cultural practice and livelihood for many Batswana farmers), and socio-political factors (i.e. Feedlot cattle with preferential access to EU marketing). Sustainability trade-offs between management systems are described in further detail below.

Free-range cattle were significantly thinner than Feedlot cattle by 53 kg, while Feedlot cattle had higher incidences of lung, liver, and spleen condemnation. It is not surprising that Free-range cattle were thinner and had fewer ‘good’ conformation carcasses than Feedlot, where the explicit production goal is to raise and fatten animals to slaughter weight in short periods of time, relying on imported concentrate feeds (corn, soybean) to do so. Free-range management, on the other hand, utilizes native pasture systems to reduce feed costs. In Botswana, communal pasture quality has been declining for some time, the result of overgrazing and climate change affecting rainfall patterns (Mogomotsi et al. 2020). Significant variability in rainfall patterns has been reported over the last decade, affecting the livelihoods of those dependant on rainfed agriculture (Kgosikoma et al. 2012, 2015; Mogotsi et al. 2013). It is therefore unsurprising that Free-range cattle were thinner at slaughter, as they experienced the consequences of climate change and management impacting pasture quality.

Differences in fat colour and composition between Free-range and Feedlot cattle can also be attributed to management. Studies have shown grass-fed cattle to have fat that is more yellow in colour (Leheska et al. 2008), with evidence that grain feeding through Feedlot causes changes in fat colour, becoming more white coloured over feedlot feeding time (Strachan et al. 1993). Differences in diet contribute to the different fat colour profiles between Feedlot (white fat) and Free-range cattle (yellow/cream fat), while the lack of fat in Free-range cattle can be attributed to severe undernutrition from poor pasture quality.

Feedlot cattle had higher rates of red offal condemnation (lung, liver, and spleen), another consequence of management differences. Close conditions of animals in feedlot management system increase the cattle’s susceptibility to a variety of infectious diseases, as well as increase in stress levels allowing subclinical illnesses opportunity to proliferate. High levels of respiratory disease across feedlot cattle were seen, as 72% of feedlot cattle sampled in the study had condemned lungs after inspection (18 of the 25 feedlot cattle). Similarly, 40% and 16% of feedlot cattle sampled had condemned livers and spleens, respectively, after inspection. The liver and spleen serve detoxification roles, so it can be theorized that feedlot animals have some level of clinical and/or subclinical disease. Free-range cattle, on the other hand, had higher levels of green offal (small and large tripe) condemnation with 36% (8/22) condemned predominately due to parasites, a management consequence of feeding on pasture.

The sole reason for whole carcass detention across management systems was infestation with beef measles (Cysticercus *bovis*). Beef measles is endemic to Botswana with national prevalence rates of 6.2% and significant variation in prevalence across districts (Mazhani et al. 2022). Bovine cysticercosis is caused by the larval stage of the human parasitic tapeworm, *Taenia saginata*. Thus, *C. bovis* is of major concern both for public health and economic profitability for the beef sector (Mazhani et al. 2022). Carcasses with more than ten cysts are condemned. Carcasses with less than ten cysts are detained and undergo cold treatment before being passed as fit for human consumption. In addition, the presence of cysts downgrades both carcasses and offal at a cost to the farmer and abattoir alike.

Food sovereignty is the right of all peoples to culturally appropriate, healthy, and nutritious foods, as well as the right of ownership of agricultural production systems (La Via Campesina 2008). Under this approach, the values, needs and culture of Botswana determine how to prioritize efforts to improve agricultural production and food availability across the country. With this in mind, major trade-offs between Feedlot and Free-range cattle management were condensed to show strengths, weaknesses, opportunities, and threats for each system. This analysis compliments the sustainability conceptual framework by providing specific details on how sustainability trade-offs affect beef management systems in Botswana.

Notably, these trade-offs are impacted by global economic markets and political systems, as evidenced by the trade-offs around imported or native feeds. Free-range cattle had smaller carcass weights with higher rates of small and large tripe condemnation, due to both parasites and declining native pasture qualities. Both Feedlot and Free-range cattle management systems are affected by climate change consequences like heat stress. Of more concern to Free-range management is drought, which deteriorates pasture and forage quality and with increased periods of high heat with minimal rainfall can lead to bushfires. However, other land use changes, including protected lands of approximately 45% of the country’s total land mass, and mining related changes impact farming resiliency strategies for both Feedlot and Free-range, as all farming systems depend on land access (Convention on Biological Diversity 2024). The SWOT analysis compliments the sustainability framework by considering how feed availability impacts cattle productivity and acknowledging that each system has its own distinct challenges, namely pasture quality degradation for free-range cattle and reliance on imported grains and feed for feedlot raised cattle.

Importantly, both management systems are impacted by climate change, which highlights the need for the research community to contribute to strategies for beef resilience. While this paper does not suggest explicit policy or farming practice recommendations, it provides a conceptual framework for sustainable livestock production in Africa, identifies evidence-based determinants of meat quality for Feedlot and Free-range cattle under Botswana conditions, and further presents the outcomes of SWOT analysis of beef cattle farming in Botswana. These findings should help relevant stakeholders (farmers, policymakers, agricultural extension, researchers, and professionals) make informed decisions regarding investment, management, policy, and programs for the betterment of Botswana agriculture. For Feedlot systems, future research should address reducing animal disease and subsequent organ condemnation rates, as well as reducing volatility in feed importation through strategies to bolster domestic and/or regional feed value chains and production. Research on Free-range systems can address pasture management and stewardship strategies, including land tenure, such that cattle reach comparable slaughter weights to feedlot counterparts. Pasture resiliency to climate change will be of growing concern for Free-range cattle management. This research should examine factors impacting sustainable agricultural implementation at scale, including socio-political sustainability factors including political will, financing, support, expertise, and research systems.

## Conclusions

Feedlot compared to Free-range cattle management reveals productivity and economic trade-offs, with Feedlot systems producing higher carcass weights and higher liver, lung, and spleen condemnation, compared to Free-range systems with lower carcass weights and lower liver, lung, and spleen condemnation. This study shows that agricultural sustainability is multifactorial, with issues such as animal productivity, animal disease burden, and beef sector profitability all existing on an interactive continuum, creating trade-offs dependent on the management system. The trade-offs become clear when the cattle farming systems are subjected to mixed-method approaches and a SWOT analysis, exposing divergent future sustainability research needs across the two management systems. Feedlot management will benefit from research on reducing animal disease and reducing imported feed dependencies whereas Free-range management requires research on pasture stewardship, land tenure, and climate change resiliency strategies.

## Supplementary Information

Below is the link to the electronic supplementary material.

Supplementary file1 (XLSX 18 KB)

## Data Availability

The datasets generated and analyzed during this study are available for public access in the Supplementary Information.
